# Trace Element Status (Iron, Zinc, Copper, Chromium, Cobalt, and Nickel) in Iron-Deficiency Anaemia of Children under 3 Years

**DOI:** 10.1155/2014/718089

**Published:** 2014-02-26

**Authors:** Maria Georgieva Angelova, Tsvetelina Valentinova Petkova-Marinova, Maksym Vladimirovich Pogorielov, Andrii Nikolaevich Loboda, Vania Nedkova Nedkova-Kolarova, Atanaska Naumova Bozhinova

**Affiliations:** ^1^Department of Chemistry and Biochemistry & Physics and Biophysics, University of Medicine-Pleven, 1 Kliment Ohridski Street, 5800 Pleven, Bulgaria; ^2^Department of Pediatrics, University of Medicine-Pleven, 1 Kliment Ohridski Street, 5800 Pleven, Bulgaria; ^3^Department of Hygiene and Ecology, Sumy State University, Medical Institute, 31 Sanatornaya Street, Sumy 40007, Ukraine; ^4^Department of Pediatrics with Medical Genetics, Sumy State University, Medical Institute, 31 Sanatornaya Street, Sumy 40007, Ukraine

## Abstract

*Aim*. To determine trace element status and aetiologic factors for development of trace elements deficiencies in children with iron-deficiency anaemia (IDA) aged 0 to 3 years. *Contingent and Methods*. 30 patients of the University Hospital, Pleven, Bulgaria—I group; 48 patients of the Sumy Regional Child's Clinical Hospital, Sumy, Ukraine—II group; 25 healthy controls were investigated. Serum concentrations of iron, zinc, copper, chromium, cobalt, and nickel were determined spectrophotometrically and by atomic absorption spectrophotometry. *Results*. Because the obtained serum levels of zinc, copper, and chromium were near the lower reference limits, I group was divided into IA and IB. In IA group, serum concentrations were lower than the reference values for 47%, 57%, and 73% of patients, respectively. In IB group, these were within the reference values. In II group, results for zinc, cobalt, and nickel were significantly lower (*P* < 0.05), and results for copper were significantly higher in comparison to controls. *Conclusion*. Low serum concentrations of zinc, copper, cobalt, and nickel were mainly due to inadequate dietary intake, malabsorption, and micronutrient interactions in both studied groups. Increased serum copper in II group was probably due to metabolic changes resulting from adaptations in IDA. Data can be used for developing a diagnostic algorithm for IDA.

## 1. Introduction

Under conditions of iron-deficiency anaemia (IDA), a host of metabolic changes representing adaptation mechanisms for maximizing iron delivery for erythropoiesis occur [1, 2]. There are close relations between the metabolism of different trace elements including iron based on antagonistic or synergistic interactions [3, 4]. One of known links is at the level of common intestinal transporters for iron and other divalent metals. Upregulation of their expression induced by iron deficiency (ID) predisposes to metabolic imbalances and respective changes in trace element status [1, 2]. Another known link is at the level of metal-storage proteins, metallothioneins, which bind different metals, thus acting in their storage and detoxification [5–7].

Interactions of different trace elements with iron determine the relationship between changes in trace element status in the organism and development of IDA. Increases in content of antagonistic to iron trace elements, such as cobalt, zinc, copper, chromium, and calcium, which impair iron absorption or its physiological impact, can lead to development of IDA. Deficiencies of synergistic to iron trace elements implicated in iron metabolism or processes of haematopoiesis, such as copper, chromium, nickel, sodium, and potassium, can contribute substantially to the aetiology of IDA [4].

Only 35–55% of cases of IDA in children are solely due to iron deficiency and others are associated with changes in status of multiple trace elements.

In our study, we use serum trace element concentrations as markers of trace element status in the organism.

Results published from different researchers on status of trace elements in IDA are various and often conflicting.

Most of the researchers have discovered lower serum zinc levels in subjects with IDA in comparison to nonanaemic subjects [8–11], but others have not found significant differences in serum zinc between IDA subjects and healthy controls [12–14].

In studies on copper content in serum and blood, higher [8–10, 12, 15] and lower levels [16, 17] as well as levels without significant differences [13, 14] have been discoveredin subjects with ID and anaemia in comparison to nonanaemic and iron-adequate subjects. Both low and high serum copper concentrations have been observed in a subset of anaemic participants in a study of Knovich et al. [18].

Although chromium is considered synergistic to iron [4] and some researchers have found lower concentrations in blood of anaemic patients when compared to control subjects [17], it is known as an antagonistic competition between trivalent chromium and trivalent iron for binding to apotransferrin [4, 19]. On the basis of this interaction, Lukaski et al. have suggested adverse effect of high-dose and long-term chromium supplementation on iron metabolism and status in adults [20].

Cobalt and nickel are essential trace elements with significant impact on the processes of haematopoiesis—stimulation of erythropoietin production and haemoglobin synthesis [21]. Lower concentrations of nickel have been observed in blood of anaemic children as compared to healthy controls [17]. Higher concentrations of cobalt have been found in blood at low body iron stores [2].

Our literature search shows that many researchers do not explain changes in trace element status with mechanisms for transport and storage.

The aim of the study is to determine trace element status, aetiologic factors, and mechanisms for development of trace elements deficiencies in children with IDA from 0 to 3 years of age.

## 2. Clinical Contingent and Methods

Our investigation comprises 78 patients from 0 to 3 years of age with clinical and laboratory signs of IDA. 30 children-patients are of the University Hospital, Medical University, Pleven, Bulgaria—I group, and 48 are patients of the Sumy Regional Child's Clinical Hospital, Sumy, Ukraine—II group. Comparison group includes 25 healthy children at the same age.

Anaemia was defined according to the criteria adopted by the WHO. Haemoglobin level below 110 g/L and haematocrit value below 0.33 l/l were used as diagnostic limits of anaemia. In I group of patients, measures of iron status, especially serum iron concentrations below 8.0 *μ*mol/L and transferrin saturation (TS) below 16%, and low red cell indices were used to identify that anaemia was due to ID [22]. Percent TS was calculated as a ratio of serum iron and total iron-binding capacity (TIBC)—serum iron/TIBC × 100. Serum ferritin values were used as indicators for iron deficiency in II group.

All children were enrolled in the study after informed consent from their parents or guardians. Ethical approval was obtained from the Institutional research ethics committees.

A parental questionnaire was provided to collect information about feeding patterns.

Fasting venous blood samples were obtained for analysis in the morning from all children into sterile tubes untreated with heparin, EDTA, citrate, and so forth. After two hours standing and centrifugation at 3500 rpm for 10 minutes, blood serum was separated. The serum samples were put in closed plastic laboratory vessels and stored at −18°C until trace element analysis.

In I group, serum content of trace elements iron, zinc, copper, and chromium was determined spectrophotometrically: ferrozine method [23] for serum iron and total iron-binding capacity by COBAS INTEGRA 400 (Roche) analyzer, spectrophotometric methods using GIESSE diagnostics (Italy) tests for serum zinc and AUDIT diagnostics (Ireland) tests for serum copper, and spectrophotometric method [24] with our modifications for serum chromium. Serum concentrations of zinc, copper, and chromium were determined by a spectrophotometer DR2800 (Hach Lange, Germany).

The serum ferritin levels were determined by ELISA using a kit of reagents “UBI MAGIVEL FERRITIN” produced by “United Biotech Inc.” (USA).

Haematological parameters, such as haemoglobin (Hb), haematocrit (Ht), red blood cell (erythrocyte) count (RBC), and the red cell indices, mean corpuscular volume (MCV), mean corpuscular haemoglobin (*МСН*), mean corpuscular haemoglobin concentration (*МСН* *С*), and red cell distribution width (RDW) were examined by analyzer MIKROS—18 (ABX). The reticulocyte count was determined by microscopic examination of a peripheral blood smear stained with a supravital dye.

Serum trace element concentrations and haematological parameters in I group of patients were compared to their respective reference values indicated in Table 1.

In II group, the content of trace elements iron, zinc, copper, cobalt, and nickel in blood serum and erythrocytes was determined by atomic absorption spectrophotometry (AAS) on a spectrophotometer C-115 M1 (JSC “Selmi,” Ukraine) [25, 26]. All results from trace element analysis and investigated haematological parameters in II group of patients were compared to healthy controls. Content of trace elements in blood serum and erythrocytes in comparison group was determined by AAS.

Statistical data processing was performed using Excel (Microsoft Corporation, Redmond, WA), Statgraphics Plus (Manugistics, Rockville, MD), and Statistica 6.1 (StatSoft, USA). All values were expressed as mean ± standard deviation (SD). Student's *t*-test and Wilcoxon's test were used to evaluate differences between study groups. Statistically significant differences were indicated by *P* values < 0.05.

## 3. Results

Clinical manifestations of IDA in all children were demonstrated by the presence of sideropenic and anaemic syndromes.

Anaemic syndrome is manifested by such symptoms as pallor of skin and mucous membranes, fatigue and faintness, tachycardia, and systolic murmur. In a number of patients apathy, drowsiness, or conversely, excessive irritability, and emotional lability were observed due to decreased oxygen delivery to the brain [36] and deficiency of iron which has been shown to play a key role in brain functions [22].

**Figure 1:**
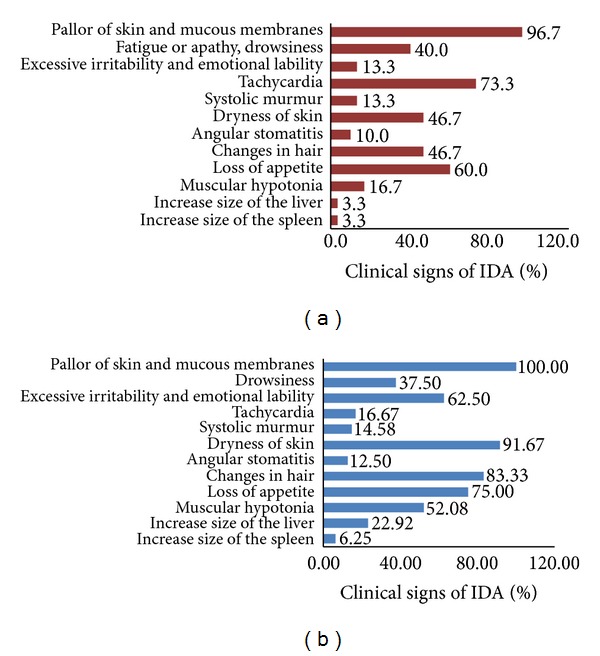
Clinical signs of IDA in I group (a) and II group (b).

Manifestations of hyposiderosis were due to deficiency of iron-containing enzymes. Dryness of skin, changes in hair—fragility, and dim color were observed; signs of angular stomatitis and atrophic glossitis were also found. Most children suffered from loss of appetite. A number of patients had a syndrome of muscular hypotonia. In some of the patients with IDA increased size of the liver and spleen was observed due to extramedullary haematopoiesis (Figures 1(a) and 1(b)).

Results of investigated clinical laboratory indicators in patients with IDA, comparison group, and the respective reference values are presented in Table 1.

**Figure 2:**
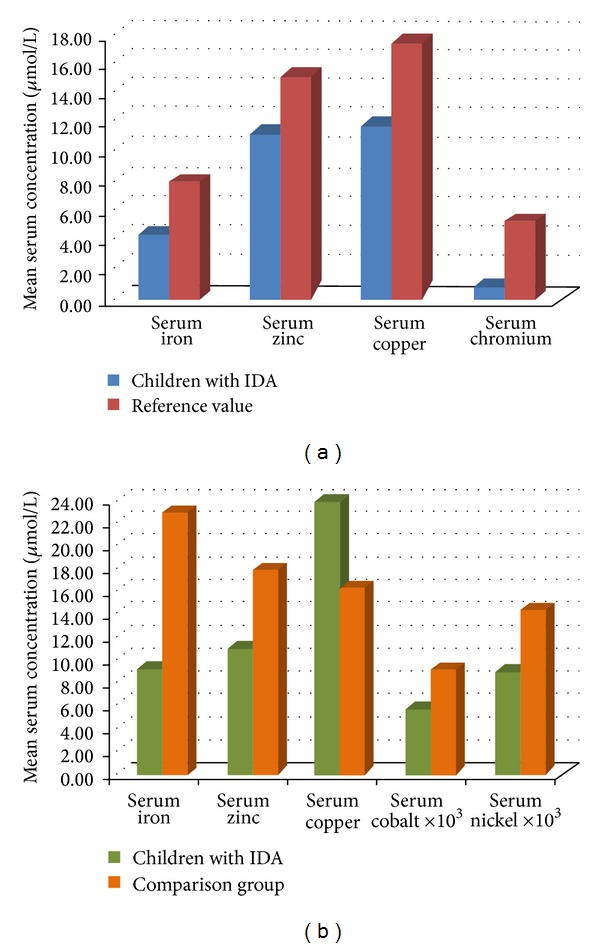
Serum concentrations of trace elements in I group (a) and II group (b) of children with IDA.

All haematological parameters in anaemic children exhibited changes in accordance with the presence of IDA. Mean value of serum iron in I group of patients with IDA was found to be lower than the reference values−4.43 ± 1.21 *μ*mol/L (Table 1, Figure 2(a)), and along with the low transferrin saturation (TS)−6.23 ± 2.65%, indicated presence of ID. In II group, serum ferritin content was found to be 9.42 ± 0.75 ng/mL which is significantly lower (*P* < 0.001) in comparison to healthy controls −38.67 ± 4.18 ng/mL.

Mean values of serum zinc, copper, and chromium in I group were all near the lower limits of the reference ranges (Table 1, Figure 2(a)). In II group of patients with IDA, mean serum iron, zinc, cobalt, and nickel concentrations were found to be significantly lower, and mean serum copper level was found to be significantly higher in comparison to their respective controls (Table 1, Figure 2(b)) with reliability level *P* < 0.05.

**Figure 3:**
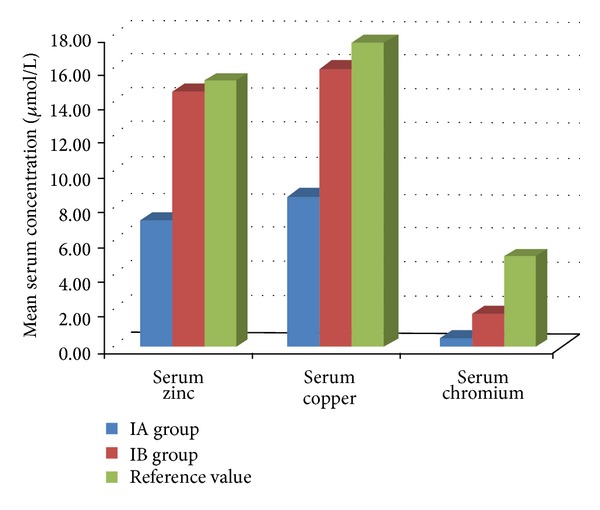
Serum concentrations of zinc, copper, and chromium among IA group and IB group of children with IDA.

In I group, results for serum levels of zinc, copper, and chromium (Table 1, Figure 2(a)) enable to divide examined patients into two groups for each of the investigated trace elements (Figure 3).

Patients with serum trace elements concentrations lower than the reference values—serum zinc 7.29 ± 2.54 *μ*mol/L, copper 8.6 ± 1.46 *μ*mol/L, and chromium 0.47 ± 0.14 *μ*mol/L, are included in IA group. For each of the investigated trace elements, the number of patients in this group constitutes 47% (*n* = 14), 57% (*n* = 17), and 73% (*n* = 22) of total number of children with IDA in I group.

Patients with serum trace elements concentrations within the reference values—serum zinc 14.65 ± 2.21 *μ*mol/L, copper 16.0 ± 3.2 *μ*mol/L, and chromium 1.83 ± 0.61 *μ*mol/L, are included in IB group. For each of the investigated trace elements, the number of patients in this group includes 53% (*n* = 16), 43% (*n* = 13), and 27% (*n* = 8) of total number of participants with IDA in I group.

There are statistically significant differences between IA and IB groups (*P* < 0.001).

Examination of trace element content in erythrocytes showed significantly lower values for all investigated trace elements in patients with IDA in comparison to control subjects (Table 2).

## 4. Discussion

Under conditions of IDA, trace element status in the organism is largely influenced by metabolic interactions between trace elements, some of which result from adaptation mechanisms for maximizing iron delivery for erythropoiesis [1, 2, 22]. Nutrition, physiologic features in different life periods, and underlying pathological conditions also affect trace element status. It has been shown that children in infancy and early childhood are particularly susceptible to deficiencies of iron and zinc, and copper deficiency occurs mainly in infancy. This vulnerability is due to increased requirements for rapid growth which are frequently not met by the diet [6, 17, 22, 36, 37].

IDA often shows association with low serum zinc levels, as well as zinc deficiency states [8–10]. In our study, obtained values for serum zinc in patients with IDA were also lower in comparison to reference values and controls (Figures 2(b) and 3(a)). These changes in zinc status are frequently explained by coexisting deficiencies of iron and zinc due to common dietary sources of both micronutrients and decreasing their intestinal absorption by the same dietary factors [9, 11].

Lower serum levels than the reference values were also obtained for copper among most patients in I group (57%, *n* = 17).

In our research, we found a number of factors associated with low serum concentrations of zinc and copper in children with IDA.

In 20% of children with IDA from I group, there was a history of preterm birth or low birth weight which are important contributing factors for zinc and copper deficiencies because of the inadequate prenatal stores and elevated requirements for growth [6, 38].

Association between short duration of breast-feeding, exclusive cow's milk feeding and low serum levels of zinc was observed in 57.14% of patients in IA group. Association between the same dietary factors and low serum levels of copper was observed in 64.7% of patients in IA group. This relationship may probably be due to the lower zinc and copper bioavailability from cow's milk in comparison to human milk and the low copper content of cow's milk [5–7, 38, 39].

Malabsorption due to cow's milk protein-induced enteropathy may be regarded as a factor for development of micronutrient deficiencies in 10% of patients with IDA [5, 6, 18, 37].

Low serum concentrations of zinc and copper in some of the investigated children may be attributed to the inadequate consumption of foods with high bioavailability of zinc and copper—meat, poultry, and fish, which are important dietary sources of zinc and copper in children's diet [5, 15, 39, 40]. Other dietary factor is the early introduction and high intake of flour-based foods containing the inhibitors of zinc and copper absorption phytates [7, 17]. These dietary factors were observed in 78.6% of patients with low serum levels of zinc and in 76.47% of patients with low serum levels of copper from I group.

The proposed mechanisms explaining low serum zinc and copper levels in some of the investigated children are antagonistic interactions between zinc and copper within the enterocyte [7]. Impaired intestinal absorption of zinc is observed under conditions of high intake of copper attributed to competitive antagonism between both metals for absorption sites in the gastrointestinal tract [3, 5].

In relatively high dietary intake of zinc, production of metal-binding proteins metallothioneins in intestinal mucosa is induced. As metallothioneins have a greater affinity for copper than zinc, this is followed by sequestration of high proportion of dietary copper in a stable copper-metallothionein complex in intestinal mucosal cells—“mucosal block” in copper transport, reduction in copper absorption, and increased copper excretion [5, 7].

Low serum concentrations of zinc and copper, which we found in investigated children, may be considered as contributing factors for IDA due to known synergistic interactions of both trace elements with iron—participation of zinc in haemoglobin synthesis and its essentiality in erythropoiesis [7, 41], and implication of copper-containing enzymes ceruloplasmin and hephaestin, ferrochelatase and cytochrom-c oxidase in iron metabolism, formation of haemoglobin, and mechanisms of hematopoiesis [4, 6, 7, 42]. Studies in animals and humans have found that copper deficiency can lead to ID [1, 4] and IDA [6, 7, 43].

It is difficult to identify factors explaining relatively high serum levels of zinc found in some patients with IDA from IB group, as well as their relationship with development of IDA. Some studies have shown that nutritional deficiency of iron enhances the intestinal absorption of zinc suggesting the divalent metal transporter 1 (DMT1) as a common absorptive pathway for both metals and physiological basis for such an interaction [44]. Although zinc is considered antagonistic to iron [4] on the basis of absorptive competition, conflicting results have been obtained in studies evaluating effect of zinc on iron absorption, and DMT1 has been postulated as an unlikely site for competitive antagonism [7].

Discovering significantly higher serum copper in II group of patients with IDA in comparison to healthy controls and relatively high serum copper in a part of IB group may be considered as a consequence of adaptation mechanisms in ID intended to maximize iron delivery for erythropoiesis [1]. There are data for upregulating the duodenal iron transporter DMT1 which is also a physiologically relevant copper transporter and the Menkes copper ATPase (MNK protein, Atp7a) on the basolateral membrane of enterocytes under low iron conditions [1, 2, 13].

It has been shown that increased copper absorption induced by ID might contribute to IDA [12] due to known antagonistic competition between copper and iron at the level of DMT1 [4, 8]. Moreover, higher serum copper concentrations are related to high levels of serum ceruloplasmin which, because of the antagonism with zinc, lowers zinc to copper (Zn : Cu) ratio. This is known to increase hemolysis and peroxidation of erythrocytes and thereby promotes anemia [7, 43]. Therefore, higher serum levels of copper in II group of patients and relatively high although in the reference range serum levels of copper in IB group may be considered as an important contributing factor for IDA.

In addition, imbalance of erythrocyte and serum copper was observed in II group of children with IDA with significant increase of its concentration in the blood serum, but copper deficiency in red blood cells (Table 2). It is known that erythrocyte deficiency of copper impairs incorporation of iron into the haem structure [43].

The serum concentrations of chromium were found to be lower than the reference values in 73% of children with IDA in I group. Other researchers [17] have also discovered significantly lower concentrations of chromium in blood of anaemic patients when compared to control subjects. Chromium is considered synergistic to iron and its deficiency can lead to ID [4]. For the rest 27% of patients with IDA in I group, serum chromium concentrations within the reference values were found. This is explained by the fact that besides being synergistic, chromium can be antagonistic to iron due to competition for binding to apotransferrin. Significantly reduced uptake of iron by serum transferrin has been observed in the presence of chromium [4, 19]. Therefore, in these children, serum chromium levels found may be associated with negative influence of chromium on iron metabolism, thus contributing to the aetiology of IDA.

Both cobalt and nickel mean serum concentrations in our study were found to be significantly lower in children with IDA than healthy controls (*P* < 0.05). Cobalt and nickel play important roles in the processes of erythropoiesis. It has been shown that both metals stimulate erythropoietin production by activation of the transcription factor hypoxia-inducible factor 1*α* (HIF-1*α*). Cobalt influences DNA synthesis accelerating maturation of erythroid stem cells and stimulates haemoglobin synthesis [21, 45]. Nickel is considered synergistic to iron by promoting its intestinal absorption and nickel deficiency can lead to ID and anaemia [4, 17, 46]. Therefore, deficiencies of both trace elements might be a contributing factor for development of IDA in our study.

However, it is difficult to identify the contribution factors explaining these results because although serum levels of cobalt and nickel were found to be lower than the control subjects, they were within the reference values. It has been shown that the intestinal absorption of iron, cobalt, and nickel is mediated by a common transport mechanism, DMT1 [2, 47], which is known to be upregulated by ID [2, 13]. It has been found, however, that transport capacity for cobalt and nickel was lower than the iron because of a higher binding constant and lower exchange rate for both metals as compared with iron. This probably not only results in suppressed duodenal uptake of iron but also explains lower levels of cobalt and nickel which we observed in children with IDA [21].

We also found that dietary factors and malabsorption may be regarded as associated with lower serum concentrations of cobalt and nickel in our patients with IDA. Important dietary sources of cobalt are animal tissues or products, such as meat, eggs, and dairy products, which were found to be scarce in the diet of the children with IDA. High dietary intake of cow's milk, frequently observed in children of infancy and early childhood, and found as a common dietary pattern in our patients with IDA, does not obligatory provide sufficient intake of cobalt because of the known geochemical cobalt deficiency. As cow's milk has low nickel content and contains factors inhibiting nickel absorption, cow's milk-based dietary patterns observed in our study may be a possible reason for lower serum levels of nickel discovered. Intestinal malabsorption, found in certain patients with IDA, is also known as contributing to low cobalt and nickel content in the organism [48–50].

## 5. Conclusion

Through the present study including investigation of trace element status, we expand the aetiology of IDA. Data obtained can be used for developing a diagnostic algorithm for IDA.

Low serum trace element concentrations of zinc, copper, cobalt, and nickel, mainly due to inadequate dietary intake, malabsorption problems, and trace element interactions, were found in both studied groups (I and II groups) of children with IDA.

Profiles of trace elements iron and zinc in blood serum do not differ in the two examined groups by the means of two analytical methods applied. Increased serum concentrations of copper in II group in comparison to control subjects are probably due to metabolic changes resulting from adaptation mechanisms in IDA.

Dietary insufficiencies of micronutrients, as well as low concentrations of trace elements in blood serum, are common in children with IDA under 3 years of age. However, mechanisms for metabolic interactions between trace elements based on transport and storage molecules are not clearly investigated yet. Micronutrient deficiency in patients with IDA can lead to the formation of so-called related functional iron deficiency. In this case, it is considered that molecular mechanisms provided by trace elements responsible for iron absorption and transport and further included in haem structure are probably impaired. This could lead to low efficiency of the monotherapy by iron supplements.

High prevalence of nutrition-related disorders in trace element status under conditions of IDA indicates the need to develop and implement appropriate intervention strategies for prevention and control of micronutrient deficiencies—supplementation, fortification, and dietary diversification/modification.

## Figures and Tables

**Table 1:** Haematological parameters and content of trace elements in blood serum of children with IDA.

Parameter	Reference values		I group with IDA (*n* = 30)	II group with IDA (*n* = 48)	Comparison group (*n* = 25)
	Mean [27, 28]	−2 SD [27, 28]			
Haemoglobin (g/L)	120	105	90.23 ± 11.09	89.79 ± 1.23	119.15 ± 2.41
Haematocrit (L/L)	0.36	0.33	0.279 ± 0.029	0.301 ± 0.004	0.3442 ± 0.006
RBC (×10^12^ cells/L)	4.5	3.7	4.41 ± 0.65	3.58 ± 0.05	4.07 ± 0.12
Rtc (*Ğ*)	10 [28]	—	—	5.5 ± 0.87	7.86 ± 0.98
MCV (fL)	78	70	64.3 ± 9.87	74.66 ± 1.08	82.52 ± 1.17
MCH (pg)	27	23	20.93 ± 4.17	24.77 ± 0.39	29.82 ± 0.56
MCHC (g/L)	330	300	322.67 ± 18.16	323.47 ± 4.34	365.17 ± 3.6
RDW (%)	1.5–15 [29]		15.58 ± 1.56	—	—
Iron (*μ*mol/L)	8.0–24.0 [30]		4.43 ± 1.21	9.23 ± 0.86	22.92 ± 1.83
Zinc (*μ*mol/L)	11.1–19.5 [5, 31]		11.22 ± 4.40	11.02 ± 1.79	17.96 ± 1.06
Copper (*μ*mol/L)	11.0–24.0 [6, 32]		11.81 ± 4.39	23.80 ± 0.76	16.50 ± 0.71
Chromium (*μ*mol/L)	0.95–9.5 [33]		0.83 ± 0.69	—	—
Cobalt (*μ*mol/L × 10^−3^)	0.00–15.25 [34]		—	5.74 ± 0.76	9.16 ± 0.61
Nickel (*μ*mol/L × 10^−3^)	1.70–10.22 [35]		—	8.99 ± 0.868	14.35 ± 1.09

**Table 2:** Content of trace element in erythrocytes in children with IDA and healthy controls.

Trace element content (*μ*g/mg ash)	II group with IDA (*n* = 48)	Comparison group (*n* = 25)
Iron*	15.58 ± 1.13	31.56 ± 1.65
Zinc*	0.208 ± 0.013	0.260 ± 0.012
Copper*	0.176 ± 0.016	0.271 ± 0.039
Cobalt*	0.0316 ± 0.0023	0.0411 ± 0.0034
Nickel*	0.0330 ± 0.0023	0.0500 ± 0.0034

*Reliability level *P* < 0.05.
